# Applying machine learning to identify autistic adults using imitation: An exploratory study

**DOI:** 10.1371/journal.pone.0182652

**Published:** 2017-08-16

**Authors:** Baihua Li, Arjun Sharma, James Meng, Senthil Purushwalkam, Emma Gowen

**Affiliations:** 1 Department of Computer Science, Loughborough University, Loughborough, United Kingdom; 2 Robotics Institute, Carnegie Mellon University, Pittsburgh, Pennsylvania, United States of America; 3 School of Clinical Medicine, University of Cambridge, Cambridge, United Kingdom; 4 Faculty of Biology, Medicine and Health, University of Manchester, Manchester, United Kingdom; Tokai University, JAPAN

## Abstract

Autism spectrum condition (ASC) is primarily diagnosed by behavioural symptoms including social, sensory and motor aspects. Although stereotyped, repetitive motor movements are considered during diagnosis, quantitative measures that identify kinematic characteristics in the movement patterns of autistic individuals are poorly studied, preventing advances in understanding the aetiology of motor impairment, or whether a wider range of motor characteristics could be used for diagnosis. The aim of this study was to investigate whether data-driven machine learning based methods could be used to address some fundamental problems with regard to identifying discriminative test conditions and kinematic parameters to classify between ASC and neurotypical controls. Data was based on a previous task where 16 ASC participants and 14 age, IQ matched controls observed then imitated a series of hand movements. 40 kinematic parameters extracted from eight imitation conditions were analysed using machine learning based methods. Two optimal imitation conditions and nine most significant kinematic parameters were identified and compared with some standard attribute evaluators. To our knowledge, this is the first attempt to apply machine learning to kinematic movement parameters measured during imitation of hand movements to investigate the identification of ASC. Although based on a small sample, the work demonstrates the feasibility of applying machine learning methods to analyse high-dimensional data and suggest the potential of machine learning for identifying kinematic biomarkers that could contribute to the diagnostic classification of autism.

## 1 Introduction

Autism spectrum condition (ASC) is a lifelong neurodevelopmental condition characterized by deficits in social communication and social interaction as well as repetitive and restricted behaviour (American Psychiatric Association, 2013) [1]. Although ASC is primarily diagnosed by these behavioural and social characteristics, autistic individuals often present with altered motor ability such as poorer eye-hand coordination, unstable balance and unusual gait patterns [2–4].

Motor impairments have received increasing interest from the autism research community in recent years [5, 6]. Untreated motor problems can result in long-term physical, psychological, and behavioural issues in autism. For example, motor impairments cause practical difficulties with daily living skills (e.g. walking/running/dressing/writing) [7, 8], which can lead to low self-esteem and anxiety [9]. Of relevance to the current work, there is also interest in understanding whether movement characteristics can be used as diagnostic biomarkers [10].

However, motor impairments are largely under-studied and poorly characterized, since current assessment techniques such as the Movement ABC [11] do not provide quantitative analysis on the spatial and temporal nature of movements. This problem prevents advances in understanding the aetiology of motor impairment, or whether motor characteristics could be used for diagnosis. The application of advanced techniques for data analysis to the field of autistic motor control is critical for forwarding both basic and translational knowledge in this area.

In this article, we explored whether movement characteristics measured during imitation can be used to differentiate people with ASC from neurotypical controls using machine learning (ML) methods. Instead of using subjective scores obtained from behavioural exams and questionnaires, we were interested in the feasibility of using non-invasive quantitative measures to objectively quantify differences and classify autism from neurotypical controls. We introduce a data-driven approach and machine learning based methods to address some fundamental problems with regard to identifying descriptive test conditions and features from high-dimensional data and often when only a small size of samples are available.

## 2 Related work

There are a number of aspects about autistic motor control that make it a promising area to explore when looking for diagnostic biomarkers. Although the exact prevalence of motor impairments is unknown, a number of studies have shown that 70–100% of the children tested with a standard motor test battery demonstrated motor ability that was outside typical limits [12]. Furthermore, motor deficits are present from birth and persist into adulthood and evidence from a recent metanalysis comparing individuals with ASC and non-ASC groups reported a large effect size of 1.067 [3]. Therefore, motor deficits appear a common, long-lasting and reproducible finding. Many of these previous studies have used standard motor test batteries such as the Movement ABC [11], which are useful to identify children with motor difficulties, but do not provide quantitative measure on the spatial and temporal nature of the movements.

By using techniques such as motion tracking, movements can be decomposed into a vast array of rich movement parameters such as kinematics (e.g. velocity and amplitude), joint angles and limb coordination patterns. By using these large quantitative data sets it could be possible to identify movement features that are characteristic of autism and consequently form a diagnostic biomarker. For example, Guha et al. [13] used motion capture data to quantify atypicality difference in facial expressions of children with and without ASC. Six basic facial emotions were analysed using methods from information theory, statistical analysis and time-series modelling. Results indicated less complex expression producing capabilities and lower symmetry between left and right regions in the ASC compared to neurotypical group.

Data analysis of autistic motor control has been largely restricted to statistics based mean group comparisons. For instance, conventionally data analysis relies on averaging a large amount of artifact-free trials. In behavioral science studies, on the one hand we may not be able to obtain enough trials per condition; on the other hand, a large proportion of data may need to be excluded from analysis since they are outliers for a stable average [14]. Furthermore, averaging assumes the consistency of response to the stimulus which can be invalid due to habituation of individuals and in-class variability among trials and participants [15]. Finally, the standard procedure of mean group averaging can cause multi-testing problems, resulting in failure on statistic power.

Researchers have more recently focused on improving data analytic methods, particularly within the context of complex data and experimental conditions. As a powerful computational tool, machine learning has shown the potential for classification and recognition tasks in a variety of fields [16, 17]. This is because its capability of modelling complex dependence and dealing with high-dimensionality noisy data accompanied by small numbers of observations. Consequently, a number of studies have begun applying machine learning methods to investigate which neural (Chanel et. al. Stahl et al. [18, 19]) or behavioural markers [20, 21] are able to differentiate individuals with and without ASC.

Stahl et al. [18] analysed event-related potential data of eye gaze performance to discriminate infant groups at high- or low-risk for later diagnosis of autism. Computational methods using regularized discriminant functions analysis (DFA), linear discrimination analysis (LDA) and SVM were explored. The accuracy was around 64% for SVM, 61% for regularized DFA, and 56% for LDA. They highlighted the need for eliminating irrelevant variables and finding the most discriminative features prior to classification in order to increase predictive accuracy.

In Wilson et al. 2014 [22], twelve cognitive scores obtained from two subcomponents of an IQ test and ten neuropsychological experimental tasks were used to study how cognitive abilities could be useful in characterizing individuals with ASC. Significant differences between 89 ASC and 89 male controls were found on five cognitive tasks using statistical *t*-tests. Interestingly, one of these tasks was motor performance. Although it was recognized that not every variable showed a remarkable impact on each group, a SVM model was still trained using all variables so as to achieve the overall identification accuracy of 81%. It was recognized that quantitative methods to determine the most significant variables could improve the accuracy by removing outlier factors.

Using data from the Autism Diagnostic Interview-Revised and The Social Responsiveness Scale, Bone et al. [23] applied a machine learning classifier to a large group of verbal individuals with ASC and those with non-ASC disorders. A novel multiple-level SVM model was proposed, and promising classification results were reported. This work proved that ML was useful for creating robust algorithms to improve ASD screening and diagnostic methods.

Grossi et al. reported the use of specialized artificial neural networks (ANNs) to build up a predictive model based on 27 potential pregnancy risk factors in autism development [24]. The artificial neural network approach achieved 80% global accuracy selecting 16 out of 27 variables. Their work demonstrates that ANNs are able to build up a predictive model, which could represent the basis for a diagnostic screening tool. Narzisi et al. [6] proposed an artificial neural network approach to study the effects of treatment in autism spectrum disorders. The developed Auto Contractive Map is a semantic connectivity map for discovering trends and associations among variables. The proposed ANN map achieved 85% of global accuracy on finding a good correlation between some variables and treatment outcome.

To date, there have been few studies that have applied machine learning to autistic motor data. Crippa et al. [25] reported a proof-of-concept study to explore whether low-functioning children with ASC could be identified by means of kinematic analysis of a simple motor task. Fifteen ASC and 15 non-autistic children were asked to pick up a ball and drop it into a hole while their movements were recorded using a motion tracker. Seventeen kinematic parameters were extracted from the upper-limb movement and seven of these were found significant for discrimination by using Fisher discrimination ratio based technology. A maximum classification accuracy of 96.7% using SVM was reported.

Although some machine learning based methods have been applied to autism, the suitability of machine learning and the principle of choosing different algorithms need to be investigated with regard to the specific behaviour examined, as well as quality and quantity of the data obtained from individual studies [26]. In the current work, we tested the feasibility for the first time of whether kinematic characteristics recorded during *imitation* of a human movement could be used to differentiate ASC and neurotypical adults. Here, we use the term “imitation” to mean “voluntary imitation” whereby a person is asked to observe an action then to voluntarily copy that action. We chose to examine imitation as studies consistently report poorer imitation for both children and adults with ASC which is particularly apparent for imitation in the absence of any visual goals [27–29]. For example, in our previous work we asked ASC and neurotypical adults to observe then imitate a sequence of hand movements, while their movements were recorded with a motion tracker [29]. The observed hand movements could occur at two different speeds (fast, slow) and two different trajectories (low, elevated) and were either directed towards visible targets (Target condition) or to no apparent targets (No Target condition). We found that the neurotypical group more closely imitated the observed kinematics of the movement (such as duration, peak velocity, vertical amplitude) than the ASC group, but principally for the No Target conditions. However, analysis was limited to mean based statistical comparison of only five kinematic parameters, which is not possible to apply to a large set of kinematic parameters.

In this paper, we returned to this dataset and extracted a larger number of 40 kinematic parameters from eight imitation conditions. By using this large quantitative kinematic dataset, we aimed to: (1) investigate whether it is possible to use kinematic data recorded during imitation to differentiate ASC and neurotypical adults; (2) determine which kinematic parameters might be more important for differentiating groups. Statistical methods were initially attempted (Section 4.1), but failed on dealing with high-dimensional data. Machine learning based methods (e.g. Naive Bayes, principal component analysis and support vector machine) were then introduced to establish the relevance of individual parameters that characterize autism from neurotypical controls. Two optimal settings from the eight imitation conditions and nine most significant kinematic parameters from the 40 features were identified that best describe variance of participant groups. Although based on a small sample, this preliminary work resulted in a good classification accuracy of 93% (Section 4.2).

## 3 Data collection and kinematics

### 3.1 Data collection

Brief methods are presented below, but for full details please see [29]. Sixteen ASC adults and 16 age-sex-IQ matched healthy controls volunteered for the experiment. All participants received written information about the study at least 48 hours before participating and all were given the opportunity to ask questions prior to written consent being taken. The study, consent procedure and the use of data were approved by both the University of Manchester, and the NHS research ethics committees. Data used in this paper are available in S1 File for other researchers.

Data from two ASC participants was excluded due to data capture issues, leaving 14 in the ASC group. Mean age (±SD) of the ASC and control participants was 32±7.69 and 29.31 ± 8.38 respectively. Mean IQ (±SD) measured using the Wechsler Abbreviated Scale of Intelligence (WASI) was 118.5 ± 12.02 for the ASC group and 119.8 ± 11 for the control group. Age and IQ did not differ significantly between the two groups (age t(28) = 0.7, p = 0.49, IQ t(28) = -0.31, p = 0.76).

**Fig 1:**
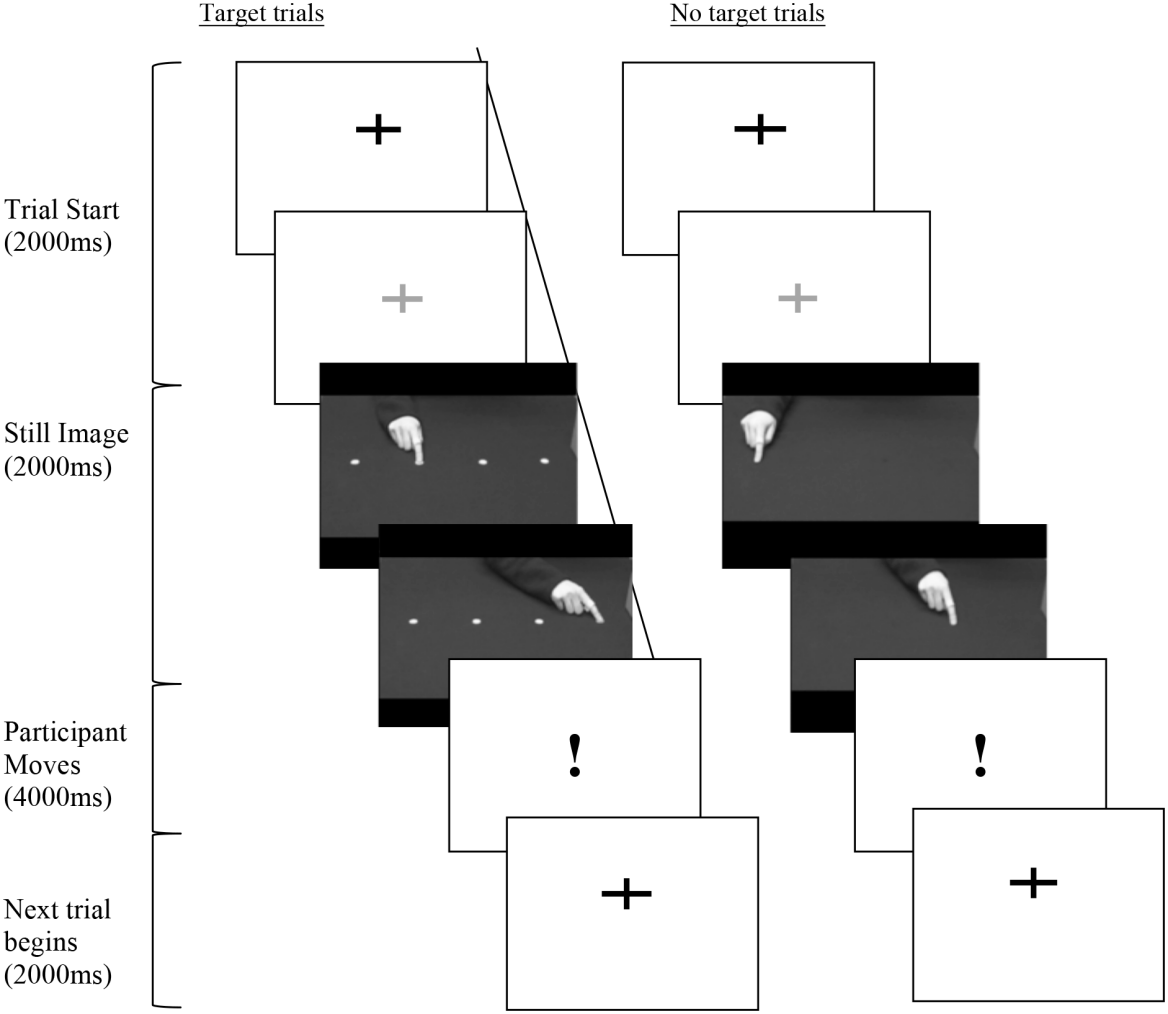
Time-lapse diagram of experiment. Each trial began with a fixation cross, followed by a still image of a hand and a video clip of a hand sequence. Participants imitated when the exclamation mark appeared.

The participants were asked to observe then imitate a sequence of hand movements, containing two movements as shown in Fig 1. The hand sequences were presented via video clips and showed a demonstrator’s hand either pointing to visible targets (T = Target) or to no apparent targets (NT = No Target). In addition, the hand movement could occur at two different speeds (F = fast, N = normal), or two different trajectories (S = short, E = elevated) as listed in Table 1. Participant movements were sampled at 120 Hz, and tracked in X, Y and Z coordinates via a motion sensor (Polhemus Liberty) attached to the index finger of the dominant hand.

**Table 1:** Four movement conditions that occurred for both the Target (T) and No Target (NT) imitation: Two different speeds (F = fast, N = normal) and two different trajectories (S = short, E = elevated).

Movement condition	Speed (beats per min)	Amplitude (cm)	Trajectory
Normal (N)	48	15	Flat
Fast (F)	58	15	Flat
Short (S)	48	10	Flat
Elevated (E)	48	15	Curved

For each participant, the experiment was split into four blocks, 2 containing the T stimuli trials and 2 containing NT stimuli trials. Trials containing the different speeds and trajectories were randomly varied in each block. Therefore, eight different movement conditions (TF, TN, TE, TS, and NTF, NTN, NTE and NTS) were examined. The same, single movement for all conditions was extracted for analysis, which occurred 8 times for each condition (64 trials per participant). To reduce predictability and prevent the participant’s behaviour becoming repetitive and stereotyped, 32 additional trial sequences were included.

### 3.2 Kinematic parameters

**Fig 2:**
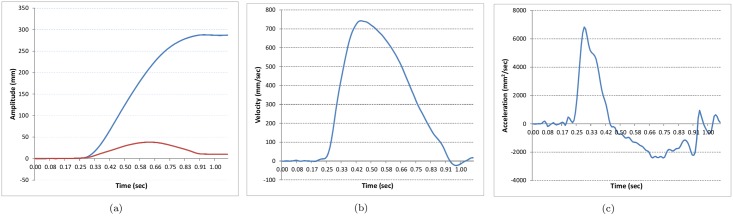
Position, velocity and acceleration traces for a rightward pointing hand movement. a) position in horizontal (blue) and vertical (red) directions. b) velocity in horizontal direction. c) acceleration in horizontal direction. Roman numerals refer to kinematic parameters in Table 2

Recorded data was analysed using Matlab. Trajectories of hand movements were filtered with a 120Hz Butterworth filter. Movement onset and offset times were determined when movement velocity rose above or fell below 10% of the peak velocity for six consecutive samples (48 ms). As listed in Table 2, twenty kinematic parameters were extracted from each trial. The mean and standard deviation (SD) of the 8 trials for each kinematic feature were calculated for each participant and for each condition. Fig 2 illustrates the meaning of position, velocity, and acceleration traces for a rightward pointing hand movement.

**Table 2:** Description of each of the 20 kinematic parameters. Roman numerals following the parameter name in the brackets indicate where referenced in Fig 2.

Kinematic parameter	Description
1.Duration (i)	Duration of movement between onset and offset (secs)
2.Peak velocity (ii)	Highest velocity (mm/sec)
3.Time to peak velocity (iii)	Time from start of movement to reach peak velocity (sec)
4.Percent peak acceleration	% of horizontal movement at which peak velocity occurs
5.Percent time before peak velocity	% of time before peak velocity
6.Percent time after peak velocity	% of time after peak velocity
7.Peak acceleration (iv)	Point of fastest acceleration (mm/sec)
8. Time to peak acceleration (v)	Time to reach peak acceleration (sec)
9.Percent time to peak acceleration	% of time to reach peak acceleration
10.Percent peak acceleration location	% of horizontal movement at which peak acceleration occurs
11.Peak deceleration (vi)	Point of fastest deceleration (mm/sec)
12.Time to peak deceleration (vii)	Time to reach peak deceleration (sec)
13.Percent time to peak deceleration	% of time to reach peak deceleration
14.Percent peak deceleration location	% of horizontal movement at which peak deceleration occurs
15.Jerk	Dimensionless jerk
16.Horizontal Max amplitude (viii)	Maximum amplitude (mm) of movement in horizontal axis
17.Vertical amplitude (ix)	Maximum height (mm) of movement in vertical axis
18.Time to peak vertical amplitude (x)	Time to reach peak vertical amplitude
19.Percent time of peak vertical amplitude	% of time to reach peak vertical amplitude
20.Percent location of peak vertical amplitude	% of horizontal movement at which peak vertical amplitude occurs

Error trials due to recording errors (e.g. the tracker failed to record) or imitation errors (incorrect sequence) led to the exclusion of 6% of trials in the ASC group and 2% of trials in the neurotypical group. Imitation errors led to the exclusion of 2% of trials in the ASC group and 1% or trials in the neurotypical group.

## 4 Methods

Using this quantitative collection of 40 kinematic parameters (means and standard deviations) under 8 imitation conditions, three questions were addressed with regard to differentiating ASC and neurotypical individuals: 1) *which imitation conditions* are suitable for differentiation; 2) *what kinematic parameters* are optimal to characterize ASC performance; 3) *what computational methods* can be used to determine such kinematic variables and achieve effective classification.

### 4.1 Statistical histogram analysis

We first performed a preliminary analysis using statistical histograms. The histogram is a plot to show the underlying frequency distribution (occurrence shape) of a set of continuous data. To construct a histogram from a continuous variable, we first split the data of a parameter into intervals, called bins. The number (or scores) of occurrences in the data set within each bin is presented using vertical bars. This method helps to describe the dataset in terms of its underlying distribution, outliers, skewness, etc.

**Fig 3:**
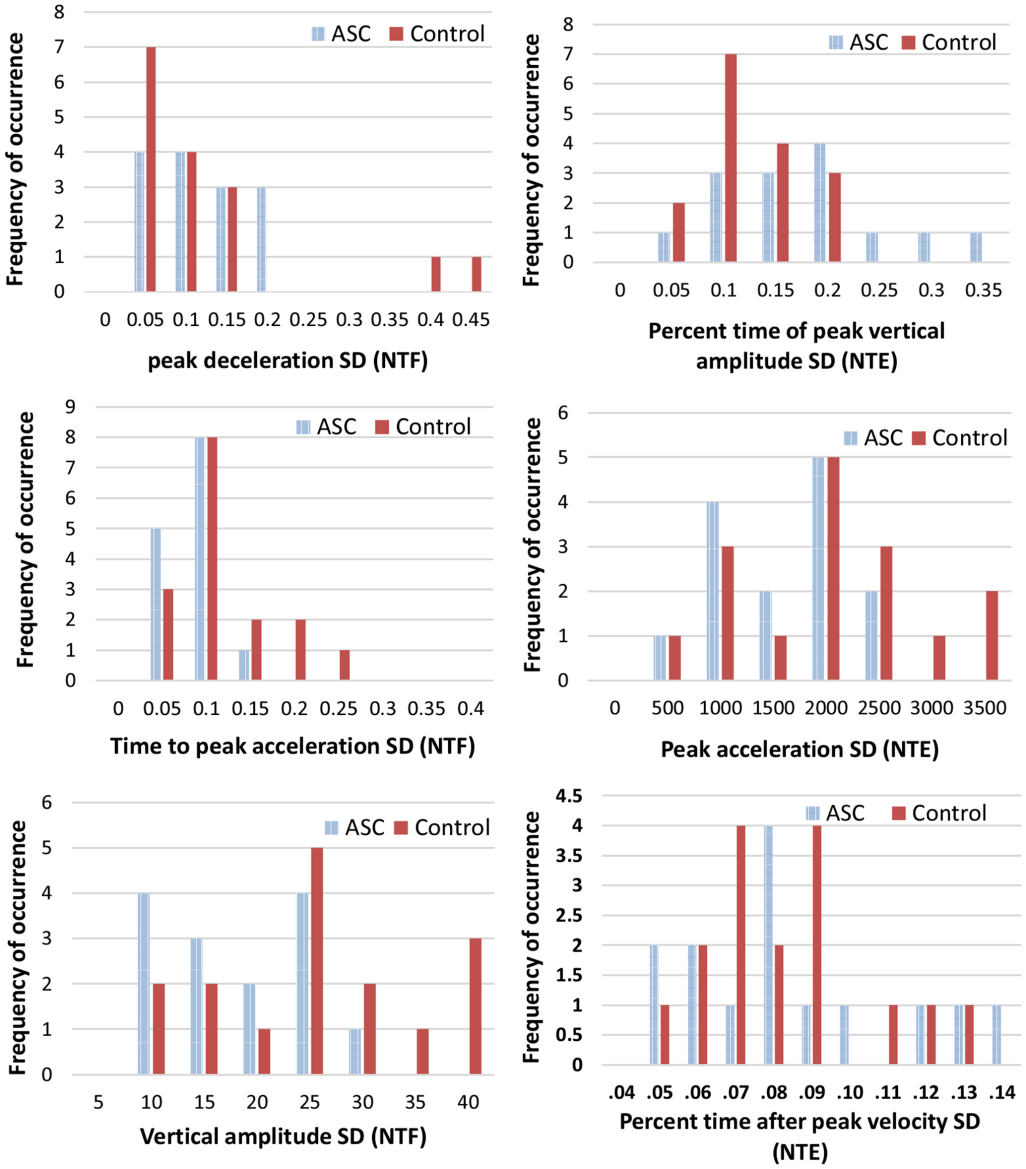
Histograms of kinematic parameters of NTF (Non-Targeted Fast, left column) and NTE (Non-Targeted Elevated, right column) experimental conditions.

The aim was to identify any obvious differences and patterns in the kinematic data between the ASC and neurotypical group across the 8 imitation conditions. To this end, histograms for each kinematic parameters at each experimental condition were generated for both ASC and control groups, and a number of statistics based comparisons (e.g. shape, density, mean, similarity distance) were conducted. Results showed that it was difficult to identify obvious patterns, sufficient agreement or large differences that could distinguish between the two groups. This can be observed in Fig 3, even for the most important parameters based on our previous work (NTF, NTE; [29]). Statistical analysis on individual parameters is not powerful enough to provide reasonable confidence on classification, especially when the dataset is relatively small. For high-dimensional features and complex conditions, methods which can effectively retrieve complicated inter/intra- relationships and dependencies from the kinematics are required.

### 4.2 Machine learning and supervised classification

Machine learning is the study of computer algorithms which can learn complex relationships or patterns from empirical data and make accurate decisions. It does not need to strictly hard code static instructions which can be difficult to obtain for complex relationships and conditions [16, 30]. Supervised learning methods are a subset of this field. The main goal of supervised classification is to learn rules from examples in different groups and use these rules to predict unseen cases into prospective classes as accurately as possible. Predictive models are built based on observed characteristics from examples in each class. The model is first trained using labelled samples. A set of input feature variables are used as input and a desired class value is used as output. The obtained classifier model is then used to recognize new samples and make a decision of their class. The reliability of the classification typically depends on the learning algorithm used by the classifier, the size and quality of the training data [17].

To expose complex relationships and dependencies among high-dimensional kinematic features, we experimented with supervised machine learning classification of varying capacities. While the limited amount of data constrains the complexity of learning models that can be used, we demonstrate that the effective use of fundamental machine learning methods can yield desirable results, for example Naive Bayes, Support Vector Machine and PCA.

Using methods explained in the following sections, models were trained to identify optimal imitation conditions, to select discriminative kinematic data, and to classify individuals as ASC or non-autistic controls. Hand kinematic data under the eight imitation conditions were examined. The accuracy of the model was evaluated by identifying unseen samples and comparing estimated labels with true memberships using cross-validation.

#### 4.2.1 Determine the best imitation conditions using Naive Bayes

We first used a Naive Bayes classifier to determine which of the 8 different imitation conditions were most suitable for discriminating ASC and neurotypical participants.

Naive Bayes model is based on probabilistic Bayes’ theorem. It is a relatively simple classifier, as it makes an assumption about the independency that each of the features are independent of the rest. While this assumption is very strong, experimentally it has been shown to achieve reasonably good results. Naive Bayes classifiers are highly scalable and fast. They take linear time rather than expensive iterative approximation as used by many other classifiers. The simplicity of the Naive Bayes allows the model to be trained with a relatively small dataset when large amounts of data samples are not available. Using Naive Bayes to identify suitable imitation conditions and significant groups of features from 40 variables with only 30 data samples prevents the overfitting problem caused by SVMs as discussed from later results.

For each imitation condition, Naive Bayes models were trained using three types of kinematic parameters as listed below. This aims not only to identify the most suitable imitation movements, but also find out the most discriminative kinematic data for autism identification.

(I) means of the 20 kinematic parameters as shown in Table 2.
(II) standard deviations of the 20 kinematic parameters
(III) both means and standard deviations of the 20 kinematic parameters

Table 3 summarizes the results from the above experiments using Naive Bayes classification models under 8 different conditions using three types of kinematic parameters. We observed that the NTE (Non-Targeted Elevated) and NTF (Non-Targeted Fast) achieved the highest average accuracies of 64.5% and 56.7%, proving to be most favorable in identifying ASC class. Meanwhile, using standard deviations for model training achieved overall better performance (average accuracy 55.4%) than using the other two types of parameters. As shown in Table 3, using standard deviations at the NTF and NTE movement conditions achieved the best classification accuracies—70.0% and 66.67%, respectively.

**Table 3:** A comparison of classification performance under eight imitation conditions and three groups of feature inputs. Accuracies I, II, III correspond to using means of 20 kinematic parameters, means of 20 standard deviations and both groups of means respectively.

Experimental setting	Accuracy (I)	Accuracy (II)	Accuracy (III)	Average
Target Fast (TF)	53.33%	50.00%	50.00	51.1%
Target Normal (TN)	43.33%	56.67%	56.67%	52.2%
Target Elevated (TE)	53.33%	60.00%	53.33%	55.6%
Target Short (TS)	40.00%	53.33%	46.67%	46.7%
Non-target Fast (NTF)	40.00%	**70.00%**	60.00%	**56.7%**
Non-target Normal (NTN)	50.00%	43.33%	43.33%	45.6%
Non-target Elevated (NTE)	60.00%	**66.67%**	66.67%	**64.5%**
Non-target Short (NTS)	50.00%	43.33%	50.00%	47.8%
Average	48.8%	**55.4**%	53.3%	

Based on the above, we attempted to train the Naive Bayes using all standard deviations of both NTF and NTE (total 40 features) simultaneously. The classification accuracy increased to 83.3%, indicating the improved performance at joint conditions of NTF and NTE. These results are very much consistent with our previous findings showing that group differences are larger for the NT conditions which, however, could not provide a quantitative comparison for the different conditions [29].

#### 4.2.2 Support Vector Machine

Having identified the optimal experimental conditions (NTF and NTE) and the standard deviations of kinematic parameters as the suitable feature category from Naive Bayes, a strong classifier Support Vector Machine (SVM) was investigated in comparison with Naive Bayes for selecting an optimal set of SD variables and achieving the best classification rate.

SVM is a supervised nonlinear multivariate classification model which possesses the ability to deal with high-dimensional datasets with complex covariance structures. However, in contrast to Naive Bayes, SVM is computationally intensive. A standard SVM attempts to find a hyperplane that perfectly separates all positive and negative samples into two non-overlapping classes.

In our experiments, different kernels (e.g. linear and RBF) and hyper-parameter settings were tested. When using the 40 standard deviations of both NTF and NTE as data features, the classification accuracy is much lower than the accuracy obtained with the Naive Bayes (83.3%). This drop in performance can be attributed to the problem of *overfitting*. Although the regularization control parameter C in SMV has been fine-tuned to prevent overfitting, it could still have an impact when a SVM model with a high-dimensional input is trained using a small dataset, as it learns to “fit” the training data perfectly, but fails on generalization. One way to deal with overfitting is to reduce input parameters by finding best ones, see Section 4.3. Our implementation of SVM was based on the LIBSVM library [31]) using Matlab. For a SVM model, the hyperplane regularization control parameters C and the *γ* when using RBF kernels need to be defined. To this end, the grid search method [32] was used to find the optimal combination (C,*γ*) in our experiments.

### 4.3 A combined method for the selection of optimal kinematic parameters

To achieve good classification accuracy, we attempted to select a subset of the most discriminative kinematic features. To determine the relative importance of the 40 kinematic parameters at NTF and NTE conditions, three methods SVM weights, leave-one-parameter-out and PCA analysis were attempted. The effectiveness of these feature selection methods was compared with three models—Navie Bayes, Linear SVM and RBF SVM. Classification performance was evaluated based on leave-one-out and using a second-layer cross-validation as described below.

Classifiers were trained and tested in leave-one-out manner. Due to the size of data is small (14 ASC and 16 controls), we used a second layer of cross-validation methodology introduced by [23], which further divided the Training data into a training/development set. At each iteration, out of the remaining 29 Training samples, we divided the set into training and development. The classifier was trained on training samples, and evaluated on the development. The performance of the classification on these development sets was used to select kinematic features and classifier parameters. Once features and parameters have been selected within the Training data, the classifier was re-trained on the entire 29 Training samples and tested on the 1 test sample in each iteration. The classification accuracy is based on the hold-out one test data of 30 iterations. Therefore we treat the test sample as unknown when selecting features and parameters. All selections are based on the performance on the Training data, not test data.

#### Method 1: SVM weights

**Fig 4:**
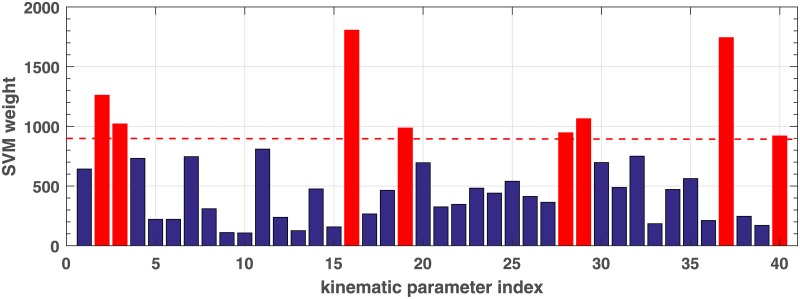
Average weights of SVMs trained on 40 NTF/NTE standard deviations showing importance of kinematic parameters. The 1*st*-20 *th* SD parameters are from NTF and the followings 20 parameters are from NTE. The parameter index are in order as shown in Table 2.

Weights of the SVM trained on 40 kinematic standard deviations of both NTF and NTE conditions were analyzed. Fig 4 illustrates the average absolute weights of trained SVMs from each iteration corresponding to each kinematic feature. The magnitude of weight indicates the importance of the kinematic parameter in the SVM classifier. We observed that some kinematic parameters contributed more on the SVM and actually consistently showed higher SVM weights at each iteration than others. Such kinematic parameters with high occurrence at each iteration include 16_*NTF*_, 37_*NTE*_, 2_*NTF*_, 29_*NTE*_, 3_*NTF*_, 19_*NTF*_ and 28_*NTE*_ etc.

According to this observation, top eight kinematic features with high contribution to SVM weights were considered as optimal features at each iteration. Classification performance of using this feature selection method on three models of RBF/linear SVMs and Naive Bayes, as shown in Table 4. The Naive Bayes achieved the asme accuracy as when using the complete set of 40 SD features (accuracy 83.3%).

**Table 4:** A comparison of results obtained using three feature selection techniques.

Method	RBF SVM	Linear SVM	Naive Bayes
SVM weights	73.3%	73.3%	83.3%
Leave-one-para-out	73.3%	76.7%	86.7%
PCA weights	63.3%	60.0%	56.7%
average	70.0%	70.0%	75.6%

#### Method 2: Leave-one-parameter-out

**Fig 5:**
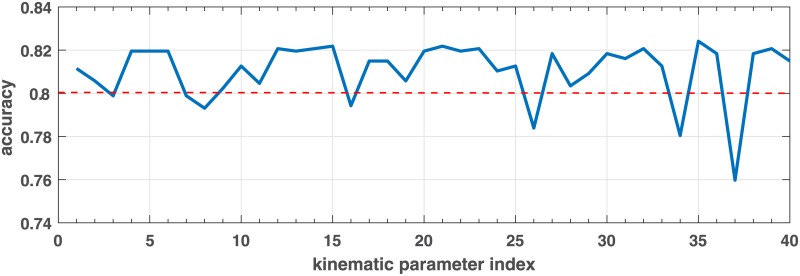
Average impact on accuracy when dropping one kinematic parameter out. The 1*st*-20 *th* SD parameters are from NTF and the followings 20 parameters are from NTE, in order as shown in Table 2.

Naive Bayes classifiers were trained by leaving one kinematic parameter out at a time on training sets. Parameters on being left out which lead to a decrease in accuracy performance below 83.3% could be more discriminative. Fig 5 shows the average impact on accuracy when leaving a corresponding kinematic feature out. Some features which on being excluded often led to a decrease in accuracy, such as 37_*NTE*_, 34_*NTE*_, 26_*NTE*_, 8_*NTF*_, 7_*NTF*_, 16_*NTF*_, 3_*NTF*_. Among these, the “vertical amplitude” 37_*NTE*_ presented a remarkable impact as shown in both Figs 4 and 5. The accuracy performance of using this selection method is shown in Table 4. The accuracy performance is competitive with the SVM-weight method as shown in Table 4, however, at each iteration kinematic features found with this method were not stable comparing to features selected by SVM-weights and PCA-based analysis.

#### Method 3: PCA weights

The principal component analysis provides a set of uncorrelated orthogonal principal components. The first principal component accounts for the largest variance in the data, and each succeeding component in turn accounts for as much of the remaining variability as possible. The contribution of each kinematic parameter to these principal components are therefore available to determine important features.

By applying PCA to 40 kinematic parameters in the training set at each iteration, the obtained average eigenvalues of the first five PCs are 0.577, 0.211, 0.149, 0.061 and 0.001 respectively. Obviously the first PC is more dominant than others, therefore contribution of each kinematic parameter to the first PC could be used for feature selection. It was found that the top several kinematic parameters with high contribution to the first PC component were very consistent at each iteration. These features are (average percentage of weight contribution to the first PC shown in brackets):

- 27_*NTE*_ (51.3%); 7_*NTF*_ (20.1%); 31_*NTE*_ (15.6%); 11_*NTF*_ (10.1%); 2_*NTF*_ (1.27%); 22_*NTE*_ (0.8%).

Selecting features using PAC-based analysis, however, resulted in a much lower classification accuracy than using features selected by the previous two methods in three models, especially when using Naive Bayes (56.6%). As shown in Table 4, the average performance on selecting 6 PCA-based features is around 60%, much lower comparing to average accuracy of 76.6% on SVM-based features (Method 1) and 78.9% on leave-one-parameter-out features (Method 2).

In summary, Table 4 shows classification accuracy performance based on feature sets selected by using each of the three methods. Features with high occurrence selected by using the three methods individually at each iteration are listed below:

- SVM weights: 2, 3, 16,19(NTF); 28, 29, 37, 40 (NTE)
- Leave-one-parameter-out: 3,7,8,16 (NTF); 26, 34, 37 (NTE)
- PCA weights: 2,7,11 (NTF); 22,27,31 (NTE)

#### Method 4: Combined feature selection

**Fig 6:**
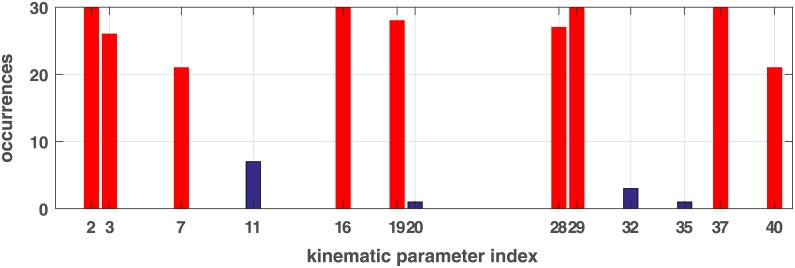
Occurrences of kinematic features selected at each iteration. The 1*st*-20 *th* parameters are from NTF and the followings 20 parameters are from NTE. The index are in order as shown in Table 2.

Feature sets selected using SVM weights are more stable and performed generally consistent on three models. Although features selected by the PCA method and leave-one-parameter-out are not optimal, some common features were deemed important. Based on above experiments, we then combined the three feature selection methods. At each test iteration, 8 SVM-weight based features and common features between top 3 PCA-based and top 10 leave-one-parameter-out features are selected as best feature set. Fig 6 illustrates the occurrences of kinematic features selected from each iteration from the training sets. The top nine features with high occurrence are:

- most discriminative kinematic features: 29,16, 37,2,19,28, 3,7,40 (Fig 6).

## 5 Autism spectrum condition classification and comparison of feature selection

To evaluate the effectiveness of these nine kinematic parameters selected by the proposed combined method above, we test the classification performance using four common classification models: Naive Bayes (NB), SVM, Decision Tree c4.5 (DT) and Random Forest (RF), as shown in Table 5. In this section, we also show comparison of our feature selection method with some standard ones in Table 6,

**Table 5:** Accuracy of five classification models on nine kinematic attributes selected using the proposed feature selection method.

Classifier	Sensitivity	Specificity	Accuracy
Naive Bayes	78.6%	81.3%	80.0%
SVM (RBF)	78.6%	87.5%	83.3%
SVM (Linear)	85.7%	87.5%	86.7%
Decision Tree	57.1%	75.0%	66.7%
Random Forest	71.4%	68.8%	70.0%
Avg.	74.3%	80.0%	77.3%

**Table 6:** Nine attribute evaluators in WEKA are used for feature selection comparison, corresponding classification accuracy (%) based on Naive Bayes (NB), SVM, Decision Tree c4.5 (DT) and Random Forest (RF) of the selected features are listed.

Attribute Evaluators	Selected Features	NB	SVM	DT	FR	Avg. (%)
Correlation Ranking	16,19,29,37,2,20,28,18,30	80	80.0	63.3	73.3	74.2
Information Gain	16,13,12,11,40,15,14,17,10	63.3	63.3	56.7	66.7	62.5
OneR Evaluator	9,20,16,29,30,8,7,37,22	83.3	80.0	66.7	73.3	75.8
ReliefF Ranking	16,37,29,19,40,1, 20,2,28	80	76.7	60.0	76.7	73.4
Symmetrical Uncertainty	16,13,12,11,40,15,14,17,19	63.3	66.7	50.0	66.7	61.7
Decision tree (DT)	29,16,28,2,8,31, 37,22,35	86.7	80.0	53.3	73.3	73.3
RandomForest (RF)	29,28,16,9,8,18,20,25,40	76.7	70.0	63.3	70.0	70.0
Logistic Regression	29, 16,1, 5,7,9,10,13,14	70	70.0	70.0	76.7	71.7
Lazy KStar	16,30, 18,29,40, 38,7,8,13	76.7	66.7	66.7	76.7	71.7
Avg. (%)		76.7	72.7	61.3	72.0	70.5

Classification performance was evaluated by means of cross-validation. Accuracy, sensitivity (True Positive (TP) rate) and specificity (True Negative (TN) rate) of classification using the proposed combined feature selection method are given in Table 5. Mathematically, they are computed as follows:

where TP (True Positive) = correctly identified, FP (False Positive) = incorrectly identified, TN (True Negative) = correctly rejected, FN (False Negative) = incorrectly rejected. Total number of autism samples (Positive) is P = 14, and total number of neurotypical controls (Negative) is N = 16. The SVM achieved a relative better result than Naive Bayes when the kinematic features are reduced from 40 to 9.

There are other methods and software tools proposed for feature selection, classification and statistical analysis of their performance [33–36]. Among these, Weka [36] is a popular software package supporting many standard data mining tasks, such as clustering, classification, feature selection and predictive modelling. It not only provides a collection of widely-used machine learning algorithms, attribute evaluators and classification models, but also model comparison through statistical cross-validation and tests. To compare our feature selection method with some standard ones [36], nine attribute evaluators in Weka were tested on our dataset, as shown in Table 6. The last 4 evaluators (DT, RF, Logistic Regression and Lazy KStar) are in the category of WrapperSubsetEval which employ a learning scheme. The top 9 features selected by each method were used for comparison based on their accuracy performance on four common classifiers. The accuracy of each model in Table 6 is the average of 10-fold cross validation.

We observe there are some similar features (e.g. 16, 29, 37) selected and highly ranked by these methods which are also similar to our features, while there are different features selected. The OneR Evaluator achieved the highest average accuracy 75.8% (STD = 8.4%) on the four models, the selected attributes include 4 significant kinematic parameters (16, 29, 37, 7) which are the same to ours. The overall accuracy of the nine feature selection methods evaluated by 4 classification models is 70.5%, while average accuracy of nine features selected by our method achieved 77.3% (STD = 8.6%)(Table 5). Based on the results in Tables 5 and 6 and statistical tests (e.g. paired T-test, cross validation) provided by Weka Experiment Environment, Naive Bayes and SVM perform better than Random Forest (RF) on average, while Decision Tree (c4.5) is almost the worst for most selected feature sets.

## 6 Discussion

In this study we investigated whether it is possible to use kinematic data recorded during imitation to discriminate between ASC and neurotypical adults and which of those kinematic features might be more important for differentiating groups. We explored an extensive set of supervised machine learning methods involving Naïve Bayes, principal component analysis and support vector machines to find the optimal imitation conditions and kinematic features for differentiating the two participant groups. Based on this preliminary study, as shown in Fig 6, a total of 9 kinematic parameters from two out of the eight imitation conditions (NTF and NTE) show significant impact:

- NTF—peak velocity SD (2), time to peak velocity SD (3), Peak acceleration (7), horizontal Max amplitude SD (16), percent time of vertical amplitude SD (19).
- NTE—time to peak acceleration SD (28), percent time to peak acceleration SD (29), vertical amplitude SD (37), percent location of peak vertical amplitude SD (40)

Best classification accuracy achieved 86.7% by using combined feature selection method with linear SVM. These results show for the first time that by using machine learning techniques on movement kinematics measured during imitation it may be possible to discriminate adults with and without ASC. We began with Naive Bayes because we were expecting overfitting with SVMs due to the nature of high-dimensional kinematic features and a limited number of data samples. The classification results show that Naive Bayes tends to perform at a similar level as SVMs even with a reduced feature set of 1/4. This may suggest that the assumption about independence among data features made by Naive Bayes could actually hold true in our case for kinematic parameters. Further study is needed to support this.

Our study demonstrates that machine learning methods could provide an effective solution to best describe motor variance of participant groups for classification tasks, while the commonly used statistical methods (e.g. mean group comparisons [29] or histogram distribution) could fail to deal with such complex experimental conditions involving high-dimensional data. Further, the proposed machine learning based methodology could help to refine experimental conditions to maximise group differences, reduce the number of trials and consequently participant time, and improve the analysis with a focus on significant features.

The only previous study combining machine learning with a motor task in autism was reported in [25]. Their machine learning methods and size of dataset are very similar. However in [25], a single, well-controlled, more informative and goal-oriented hand “activity” was examined. In addition, they focussed on selecting the features from only one test condition which reduces the complexity and computational difficulty of the problem. Although still a lab based study, our movement task was less constrained and is the first to explore the best test conditions, providing information on which activities would be most discriminating in future testing. Furthermore, instead of children, our work involved adults, where one might expect movement differences between groups to be smaller.

In line with findings from our previous work [29], two No-target conditions (No-target Elevated and No-target Fast) resulted in the highest discrimination accuracy. The results are also consistent with a large body of evidence indicating that children and adults with ASC show poorer imitation skills compared to neurotypical groups during goal-less imitation, but these differences are less apparent for goal directed imitation [27, 29]. The higher discriminative qualities of the No-target Fast and No-target Elevated conditions compared to the slower No-target normal condition may have been due to the more challenging nature of the faster and elevated trials. There is also greater requirement to make a movement that is different to the usual kinematic pattern in the faster and elevated trials [37] that may be more difficult for the participants with ASC. For example, in a recent study, neurotypical adults and those with ASC were asked to imitate the trajectory of a dot moving on a computer tablet using a hand held stylus [37]. While in some trials the trajectory was based on a typical human movement (peak velocity occurring at 50% of the trajectory), for other trials this movement was modified to produce an atypical profile (peak velocity occurring at 18% of the trajectory). In order to imitate the atypical model, participants would have to create a new sensorimotor plan to represent the novel movement kinematics, as opposed to imitating the typical model where they could re-scale an existing plan of a typical aiming movement. While the neurotypical group modified their movement kinematics to imitate the atypical profile, the ASC group did not. Therefore, to maximise group differences it is suggested that imitation studies should employ actions that require the observer to perform a dissimilar action to what might already exist within their movement repertoire.

The nine most discriminative kinematic features involved standard deviations. Interestingly, the standard deviations of all but one feature were higher in the ASC group for the No-target Fast features, but were all lower (with one exception) for the ASC group for the No-target elevated features. Increased variability is a common finding in autistic groups and has been reported for reaching movements [38], saccadic eye movements [39], and stride length and head shoulder and trunk position during walking [40]. The increased variability for the ASC group highlights differences in the ability to plan and control sensorimotor processes that is particularly apparent when attempting to imitate more challenging faster movements. In contrast, the ASC group showed lower standard deviations for the No-target elevated movements. It has been suggested that variability is more sensitive to the effects of learning [41], so it is possible that increases in variability might reflect the learning process as participants attempted to apply different strategies to the imitation task. Therefore, as discussed earlier it is likely that the neurotypical participants attempt to imitate the unusual elevated movement, increasing their variability, whereas the autistic group imitate the movement less and retain their usual style of movement. This is supported by our recent findings showing that when neurotypical participants are specifically asked to attend to and imitate the elevated movement, variability of vertical amplitude increases [42].

Overall, our work highlights a promising new approach to understanding autistic motor behaviour and diagnosing autism. The range of movements needs to be expanded to more complex, full body motor control functions, but the current results are encouraging as the analysis was based on simple movements.

This study is limited to a small sample size of participants. In the case of our small dataset, we proposed feature selection based on three methods: SVM weights, leave-one-parameter-out and PCA weights (Section 4.3). We found discriminative features detected by combining three feature selection methods produced more reliable results with reasonable bias than many standard ones. In particular, we employed a second-layer cross-validation framework for model training and feature selection ([23]). It facilitates features and model parameters are selected based on performance in the training data through a second-layer cross-validation while treats the test sample (in leave-one-out) as unknown.

As ASC is a heterogenous condition, the degree that the classification model is able to generalize to a wider population who diverge out of the average range of this dataset remains to be tested. Training the model with larger groups of ASC and controls would be helpful to ultimately improve the generalization of the model to a wide range of ASC. Similarly, we need to compare imitation in ASC with other groups that are known to have motor deficits such as Developmental Coordination Disorder and Attention Deficit Hyperactivity Disorder. Here, machine learning could be valuable in understanding whether these groups have similar motor profiles or aspects that are distinct for the different conditions as suggested by recent work [43]. It could also be useful in understanding whether there are subgroups of motor difficulties with the autistic population and consequently how these might affect different aspects of daily living with implications for therapy.

## 7 Conclusion

To our knowledge, this is the first attempt to investigate identification of autism using kinematic parameters of imitation and machine learning. We proposed the use of various machine learning techniques for the classification of autism from neurotypical controls based on movement characteristics during imitation. 40 kinematic measures from 8 different imitation conditions were analysed. It was found the NTF (Non-target Fast) and NTE (Non-target Elevated) were the most distinctive imitation conditions. Meanwhile 9 kinematic parameters were identified as the most discriminative features. SVM and Naive Bayes classifiers were implemented and compared. Although based on a small sample set, this preliminary work resulted in a good classification accuracy of 86.7% using the SVM classifier.

Given the accuracy and objective nature of feature selection and classification models, results from this exploratory study may prove valuable in demonstrating the possibility of using quantitative kinematic measures and machine learning for preliminary diagnosis of autism. In addition, we introduce methods to quantitatively identify the most significant kinematic variables that offer insight into possible motor bio-signatures, which may potentially be useful in discriminating different motor subgroups of autism.

## Supporting information

S1 File S1_File.xlsx contains the dataset used in this paper. (XLSX)

## References

[1] American Psychiatric Association, Diagnostic and statistical manual of mental disorders (5th ed.). Arlington, VA: American Psychiatric Publishing; 2013.

[2] Gowen E, Hamilton A. Motor abilities in autism: a review using a computational context. Journal of autism and developmental disorders. 2013;43(2):323–344. 10.1007/s10803-012-1574-0 22723127

[3] Fournier KA, Hass CJ, Naik SK, Lodha N, Cauraugh JH. Motor coordination in autism spectrum disorders: a synthesis and meta-analysis. Journal of autism and developmental disorders. 40(10):1227–1240. 10.1007/s10803-010-0981-3 20195737

[4] Bhat AN, Landa RJ, Galloway JCC. Current perspectives on motor functioning in infants, children, and adults with autism spectrum disorders. Physical Therapy. 2011;91(7):1116–1129. 10.2522/ptj.20100294 21546566

[5] Torres EB, Donnellan AM. Autism: The movement perspective. Frontiers Media SA; 2015. 10.3389/fnint.2015.00012 PMC4360560 25852501

[6] Narzisi A, Muratori F, Buscema M, Calderoni S, Grossi E. Outcome predictors in autism spectrum disorders preschoolers undergoing treatment as usual: insights from an observational study using artificial neural networks. Neuropsychiatric disease and treatment. 2015;11:1587 10.2147/NDT.S81233 26170671 PMC4494609

[7] Jasmin E, Couture M, McKinley P, Reid G, Fombonne E, Gisel E. Sensori-motor and daily living skills of preschool children with autism spectrum disorders. Journal of autism and developmental disorders. 2009;39(2):231–241. 10.1007/s10803-008-0617-z 18629623

[8] MacDonald M, Lord C, Ulrich D. The relationship of motor skills and adaptive behavior skills in young children with autism spectrum disorders. Research in autism spectrum disorders. 2013;7(11):1383–1390. 10.1016/j.rasd.2013.07.020 25774214 PMC4356945

[9] Skinner RA, Piek JP. Psychosocial implications of poor motor coordination in children and adolescents. Human movement science. 2001;20(1):73–94. 10.1016/S0167-9457(01)00029-X 11471399

[10] Esposito G, Pasca SP. Motor abnormalities as a putative endophenotype for Autism Spectrum Disorders. Front Integr Neurosci. 2013;7(Pt 43):1–5. 23781177 10.3389/fnint.2013.00043 PMC3678087

[11] Henderson SE, Sugden DA, Barnett AL, Smits-Engelsman C. Movement assessment battery for children. Psychological Corporation London; 1992.

[12] Hilton CL, Zhang Y, White MR, Klohr CL, Constantino J. Motor impairment in sibling pairs concordant and discordant for autism spectrum disorders. Autism. 2011;p. 1362361311423018. 10.1177/1362361311423018 PMC4222044 22013131

[13] Guha T, Yang Z, Ramakrishna A, Grossman RB, Hedley D, Lee S, et al. On quantifying facial expression-related atypicality of children with autism spectrum disorder. IEEE Int Conf Acoustics, Seech and Signal Processing (ICASSP). p. 803–807. 10.1109/ICASSP.2015.7178080 PMC4687751 26705397

[14] Stets M, Stahl D, Reid VM. A meta-analysis investigating factors underlying attrition rates in infant ERP studies. Developmental neuropsychology. 2012;37(3):226–252. 10.1080/87565641.2012.654867 22545660

[15] Stets M, Reid VM. Infant ERP amplitudes change over the course of an experimental session: implications for cognitive processes and methodology. Brain and Development. 2011;33(7):558–568. 10.1016/j.braindev.2010.10.008 21115312

[16] Mitchell TM. Machine learning. McGraw-Hill Boston; 1997.

[17] Kotsiantis SB, Zaharakis I, Pintelas P. Supervised machine learning: A review of classification techniques; 2007.

[18] Stahl D, Pickles A, Elsabbagh M, Johnson MH, Team B, et al Novel machine learning methods for ERP analysis: a validation from research on infants at risk for autism. Developmental neuropsychology. 2012;37(3):274–298. 10.1080/87565641.2011.650808 22545662

[19] Chanel G, Pichon S, Conty L, Berthoz S, Chevallier C, Grèzes J. Classification of autistic individuals and controls using cross-task characterization of fMRI activity. NeuroImage: Clinical. 2016;10:78–88. 10.1016/j.nicl.2015.11.010 26793434 PMC4683429

[20] Kosmicki J, Sochat V, Duda M, Wall D. Searching for a minimal set of behaviors for autism detection through feature selection-based machine learning. Translational psychiatry. 2015;5(2):e514 10.1038/tp.2015.7 25710120 PMC4445756

[21] Wall D, Kosmicki J, Deluca T, Harstad E, Fusaro V. Use of machine learning to shorten observation-based screening and diagnosis of autism. Translational psychiatry. 2012;2(4):e100 10.1038/tp.2012.10 22832900 PMC3337074

[22] Wilson CE, Happé F, Wheelwright SJ, Ecker C, Lombardo MV, Johnston P, et al The Neuropsychology of Male Adults With High-Functioning Autism or Asperger Syndrome. Autism Research. 2014;7(5):568–581. 10.1002/aur.1394 24903974 PMC4489335

[23] Bone D, Bishop S, Black MP, Goodwin MS, Lord C, Narayanan SS. Use of machine learning to improve autism screening and diagnostic instruments: effectiveness, efficiency, and multi-instrument fusion. Journal of Child Psychology and Psychiatry. 2016 10.1111/jcpp.12559 27090613 PMC4958551

[24] Grossi E, Veggo F, Narzisi A, Compare A, Muratori F. Pregnancy risk factors in autism: a pilot study with artificial neural networks. Pediatric research. 2016;79:339–347. 10.1038/pr.2015.222 26524714

[25] Crippa A, Salvatore C, Perego P, Forti S, Nobile M, Molteni M, et al Use of Machine Learning to Identify Children with Autism and Their Motor Abnormalities. Journal of autism and developmental disorders. 2015;45(7):2146–2156. 10.1007/s10803-015-2379-8 25652603

[26] Martin A, Volkmar FR, Lewis M. Lewis’s child and adolescent psychiatry: a comprehensive textbook. Lippincott Williams & Wilkins; 2007.

[27] Rogers SJ, Young GS, Cook I, Giolzetti A, Ozonoff S. Imitating actions on objects in early-onset and regressive autism: Effects and implications of task characteristics on performance. Development and Psychopathology. 2010;22(01):71–85. 10.1017/S0954579409990277 20102648 PMC2940421

[28] Hobson RP, Hobson JA. Dissociable aspects of imitation: A study in autism. Journal of experimental child psychology. 2008;101(3):170–185. 10.1016/j.jecp.2008.04.007 18572186

[29] Wild KS, Poliakoff E, Jerrison A, Gowen E. Goal-directed and goal-less imitation in autism spectrum disorder. Journal of autism and developmental disorders. 2012;42(8):1739–1749. 10.1007/s10803-011-1417-4 22146933

[30] Bishop CM. Pattern Recognition and Machine Learning. 2006.

[31] Chang CC, Lin CJ. LIBSVM: a library for support vector machines. ACM Trans Intelligent Systems and Technology (TIST). 2011;2(3):27.

[32] Friedman J, Hastie T, Tibshirani R. The elements of statistical learning. vol. 1 Springer series in statistics Springer, Berlin; 2001.

[33] Chandrashekar G, Sahin F. A survey on feature selection methods. Computers & Electrical Engineering. 2014;40(1):16–28. 10.1016/j.compeleceng.2013.11.024

[34] Pedregosa F, Varoquaux G, Gramfort A, Michel V, Thirion B, Grisel O, et al Scikit-learn: Machine Learning in Python. Journal of Machine Learning Research. 2011;12:2825–2830.

[35] Rodríguez-Fdez I, Canosa A, Mucientes M, Bugarín A. STAC: a web platform for the comparison of algorithms using statistical tests. In: Proceedings of the 2015 IEEE International Conference on Fuzzy Systems (FUZZ-IEEE); 2015.

[36] Frank E, Hall M, Witten I. The WEKA Workbench Online Appendix for “Data Mining: Practical Machine Learning Tools and Techniques”, 4th edn Morgan Kaufman, Burlington 2016.

[37] Hayes SJ, Andrew M, Elliott D, Gowen E, Bennett SJ. Low Fidelity Imitation of Atypical Biological Kinematics in Autism Spectrum Disorders Is Modulated by Self-Generated Selective Attention. Journal of autism and developmental disorders. 2015;p. 1–12. 26349922 10.1007/s10803-015-2588-1

[38] Nazarali N, Glazebrook CM, Elliott D. Movement planning and reprogramming in individuals with autism. Journal of Autism and Developmental Disorders. 39(10):1401–1411. 10.1007/s10803-009-0756-x 19466535

[39] Schmitt LM, Cook EH, Sweeney JA, Mosconi MW. Saccadic eye movement abnormalities in autism spectrum disorder indicate dysfunctions in cerebellum and brainstem. Molecular autism. 2014;5(1):1 10.1186/2040-2392-5-47 25400899 PMC4233053

[40] Vernazza-Martin S, Martin N, Vernazza A, Lepellec-Muller A, Rufo M, Massion J, et al Goal directed locomotion and balance control in autistic children. Journal of autism and developmental disorders. 2005;35(1):91–102. 10.1007/s10803-004-1037-3 15796125

[41] Schmidt R, Lee T. Motor control and learning: a behavioral emphasis. Hum Kinetics, Champaign; 2011.

[42] Bek J, Poliakoff E, Marshall H, Trueman S, Gowen E. Enhancing voluntary imitation through attention and motor imagery. Experimental brain research. 2016;p. 1–10. 10.1007/s00221-016-4570-3 PMC4893065 26892882

[43] Ament K, Mejia A, Buhlman R, Erklin S, Caffo B, Mostofsky S, et al Evidence for specificity of motor impairments in catching and balance in children with autism. Journal of autism and developmental disorders. 2015;45:742–751. 10.1007/s10803-014-2229-0 25231287 PMC4342267

